# Evaluation of changes in serum biochemical parameters in zarudny’s spur-thighed tortoises (*Testudo graeca zarudnyi*) influenced by gender and season

**DOI:** 10.1186/s12917-023-03862-3

**Published:** 2024-01-06

**Authors:** Zohreh Khaki, Amir Rostami, Farshad Esfandiary

**Affiliations:** 1 Department of Clinical Pathology, Faculty of Veterinary Medicine, University of Tehran, Qareeb St., Azadi Ave, Tehran, Iran; 2 Department of Internal Medicine, Faculty of Veterinary Medicine, University of Tehran, Tehran, Iran; 3 Student of Veterinary Medicine, Faculty of Veterinary Medicine, University of Tehran, Tehran, Iran

**Keywords:** *Testudo graeca zarudnyi*, Tortoise, Biochemical parameters, Season, Iran

## Abstract

**Background:**

The Zarudny’s spur-thighed tortoise or Iranian tortoise (*Testudo graeca zarudnyi*) has just been reported from Iran so far. Therefore, the purpose of this study was to determine the effects of season and gender on serum biochemical parameters of this valuable species in Iran.

**Results:**

This study was performed on 20 clinically healthy adult Zarudny’s spur-thighed tortoises. Blood samples were collected from the jugular vein and then serum biochemical parameters and body weight were measured in autumn and winter. The following biochemical parameters were measured: total cholesterol, triglyceride, high-density lipoprotein cholesterol, low-density lipoprotein cholesterol, very low-density lipoprotein, total protein, creatinine, urea, glucose, calcium, inorganic phosphorus, total bilirubin, uric acid, alanine aminotransferase, aspartate aminotransferase, alkaline phosphatase and lactate dehydrogenase. Urea concentration increased significantly at emergence from hibernation. Also, in winter, total protein, phosphorous, creatinine, total bilirubin concentrations and alkaline phosphatase activity decreased significantly compared to autumn, but aspartate aminotransferase and lactate dehydrogenase activities were significantly higher than in autumn. There was no significant difference for the parameters mentioned above by gender, except for phosphorous. Phosphorous concentration in females was significantly higher than that of males in autumn. Cholesterol and low-density lipoprotein cholesterol levels in females were significantly higher than males in autumn and winter.

**Conclusions:**

The present study is the first study that monitors the serum biochemical parameters of adult Zarudny’s spur-thighed tortoises based on season and gender. Seasonal reference intervals should be used for biochemical parameters in this valuable species. Also, sex-specific reference intervals for phosphate and cholesterol are necessary.

## Background

The Zarudny’s spur-thighed tortoise or Iranian tortoise (*Testudo graeca zarudnyi*) is terrestrial [1, 2] and native from the central Iranian Plateau to the east and southeast [1], but it also lives in other regions of Iran [3]. This tortoise has just been reported from Iran so far [4]. Also, *Testudo graeca* is currently on the International Union for Conservation of Nature (IUCN) **Red List** of **Vulnerable** and **Endangered Species** [5]. Therefore, this valuable tortoise has a high local, national and international conservation status [6].

In tortoises, it has been previously reported that species and physiologic status (such as season, sex, age, growth, and nutrition) affect blood biochemical parameters [7–11]. Also, during hibernation, changes in blood biochemical parameters may be observed due to decreased food intake and decreased metabolic activity [12]. In reptiles, laboratory tests including hematological and blood biochemical tests are used for health monitoring as in other species. Biochemical analysis of blood is an important and useful tool to finalize animal health and disease [13]. For this reason, there is a need for appropriate reference intervals for the interpretation of laboratory data in any species. However, there are no reports of serum biochemical parameters in Zarudny’s spur-thighed tortoises. Therefore, the aim of this study was to determine the effect of season (autumn and winter) and gender on the serum biochemical parameters of adult captive Zarudny’s spur-thighed tortoises in Iran.

## Methods

In this study, twenty adult spur-thighed tortoises (*Testudo graeca zarudnyi*), 10 females and 10 males, were kept in an enclosure in Tehran Zoo, approximately northwest of Tehran. This study was conducted in accordance with the animal ethical guidelines of the faculty of veterinary medicine, University of Tehran, Tehran, Iran (No: 7508017.6.26). Tortoises were clinically healthy and in good condition. The clinical examination included general health assessment (activity level, appetite and stool status) and shell observation (form and strength of the carapace and plastron) as well as observation of the eyes, mouth, nose, skin, extremities and tail. Additionally, a blood smear was prepared from each of the tortoises. In the examination of blood smears stained with Giemsa, it was confirmed that the tortoises did not have blood parasites. They were fed daily with green leaves and grass, cabbage, lettuce and dandelion, ad libitum. The diet was sometimes supplemented with carrots, strawberries and potato. Hibernation of the tortoises occurs from late November to February/March when temperatures are less than 8 °C (50 °F ), in special boxes filled with dry foliage and bark mulch. Before each sampling, body weight was measured and recorded, and then blood samples (approximately 2 ml) were collected from the Jugular vein in autumn (early November 2022, before hibernation) and winter (late February 2022, at or just prior to emergence from hibernation). Blood samples were immediately transferred into tubes without any anticoagulant for serum separation. To prepare serum, samples were clot (at 8 °C, until 1 h) and then centrifuged at 2500 rpm for 10 min. Then serum was transferred to individual tubes and biochemical parameters were immediately measured using commercial kits (Biorexfars, Tehran, Iran) and an automated chemistry analyzer (Selectra Prom, Elitech group, France). The following biochemical analytes were measured: total cholesterol (CHOD-PAP method), triglyceride (GPO- PAP method), high-density lipoprotein cholesterol (HDL-C)(enzymatic method), total protein (biuret method), creatinine (modified Jaffe method), urea (urease method), glucose (GOD-PAP method), total calcium (Arsenazo III method), inorganic phosphorus (Molybdate method), total bilirubin (Jendrassik Grof method), uric acid (uricase method), alanine aminotransferase (ALT), aspartate aminotransferase (AST), alkaline phosphatase (ALP) and lactate dehydrogenase (LDH) (enzymatic method). Also, cortisol (Elisa kit, Competitive EIA, Cat. No.2924-96) was measured using commercial kit (Ideal Diagnostic Atieh, Tehran, Iran). In addition, very low-density lipoprotein (VLDL) was estimated by one-fifth of triglyceride, and low-density lipoprotein cholesterol (LDL-C) was calculated by Friedewald’s formula: LDL-C = Total cholesterol-(HDL + VLDL) [14]. For quality control, two levels of control material were analyzed along with serum samples [15]. For statistical analysis, normal distribution of the data was confirmed by the Kolmogorov- Smirnov test. Then data were analyzed by t-student test (SPSS.22, Chicago, USA), and *P* < 0.05 was considered significant. Also, data are described as means ± standard deviation (SD).

## Results

The seasonal changes of serum lipid profile and body weight in clinically healthy adult Zarudny’s spur-thighed tortoises are shown in Table 1. Table 2 also shows the seasonal changes of some serum biochemical parameters, and the seasonal changes of glucose, cortisol and enzyme activities in clinically healthy adult Zarudny’s spur-thighed tortoises are shown in Table 3.

**Table 1:** Seasonal changes of serum lipid profile and body weight in clinically healthy adult Zarudny’s spur-thighed tortoises

	Autumn (mean ± SD)	Winter (mean ± SD)	*P* value
Body weight (g)	1863.8 ± 387.72 (1205–2600)	1805.6 ± 416.32 (1106–2640)	0.650
Total Cholesterol (mg/dl)	154.45 ± 66.41 (85–323)	133.8 ± 62.10 (59–339)	0.316
Triglycerides (mg/dl)	58.8 ± 19.26 (27–61)	61.85 ± 45.90 (8-189)	0.786
LDL-C (mg/dl)	86.96 ± 68.83 (6.1–238/7)	65.6 ± 61.62 (12.1-295.8)	0.308
HDL-C (mg/dl)	53.73 ± 24.97 (15–93)	56.23 ± 13.72 (36–87)	0.697
VLDL-C (mg/dl)	11.76 ± 3.85 (5.4–18.2)	11.70 ± 9.40 (1.6–37.8)	0.980

**Table 2:** Seasonal changes of some biochemical parameters in clinically healthy adult Zarudny’s spur-thighed tortoises

	Autumn (mean ± SD)	Winter (mean ± SD)	*P* value
Urea (mg/dl)	18.35 ± 2.75 (13–22)	87.05 ± 23.11 (64–159)	< 0.001*
Creatinine (mg/dl)	0.25 ± 0.05 (0.2–0.3)	0.18 ± 0.07 (0.1–0.3)	0.002*
Total protein (mg/dl)	4.44 ± 0.75 (3-6.06)	3.89 ± 0.71 (2.89–5.3)	0.024*
Total Bilirubin (mg/dl)	0.07 ± 0.01 (0.05–0.09)	0.04 ± 0.00 (0.03–0.07)	< 0.001*
Uric acid (mg/dl)	1.68 ± 0.30 (1/2-2.1)	1.66 ± 0.61 (1-3.1)	0.890
Calcium (mg/dl)	10.14 ± 1.04 (8.4–13.2)	10.02 ± 0.96 (8.8–11.7)	0.693
Phosphorus (mg/dl)	2.10 ± 0.28 (1.37–2.64)	1.56 ± 0.30 (1.12–1.98)	< 0.001*

*-Significant

**Table 3:** Seasonal changes of glucose, cortisol and enzyme activities in clinically healthy adult Zarudny’s spur-thighed tortoises

	Autumn (mean ± SD)	Winter (mean ± SD)	*P* value
Glucose (mg/dl)	51.30 ± 13.22 (40–98)	59.25 ± 12.89 (30–85)	0.062
Cortisol (µg/dl)	3.25 ± 0.57 (2.11–4.33)	3.36 ± 1.02 (1.98–5.26)	0.762
ALT (U/L)	23.70 ± 5.00 (14–32)	25.65 ± 12.43 (6–43)	0.519
AST (U/L)	49.20 ± 7.69 (37–71)	109.05 ± 44.11 (48–221)	< 0.001*
ALP (U/L)	392.5 ± 131.57 (121–630)	260.9 ± 84.02 (178–445)	0.001*
LDH (U/L)	234.1 ± 64.14 (135–420)	334.6 ± 90.70 (207–487)	< 0.001*

*-Significant

Although the mean weight, serum cholesterol and LDL-C levels were lower in winter, these changes were not significant (*P* = 0.650). Also, serum triglycerides, VLDL (*P* = 0.980) and HDL-C (*P* = 0.697) concentrations did not differ significantly between seasons.

Our results showed that the urea levels increased significantly (approximately 4.7-fold) at emergence from hibernation (*p* < 0.001). Also, in the winter, the concentration of serum total protein (*p* = 0.024), phosphorous (*p* < 0.001), creatinine (*p* = 0.002) and total bilirubin (*p* < 0.001) as well as ALP activity (*p* = 0.001), decreased significantly compared to autumn, but serum AST (*p* < 0.001) and LDH (*p* = 0.044) activities were significantly higher than in autumn. Although an increase in serum glucose was observed in winter compared to autumn, these changes were not significant (*p* = 0.062).

**Fig. 1:**
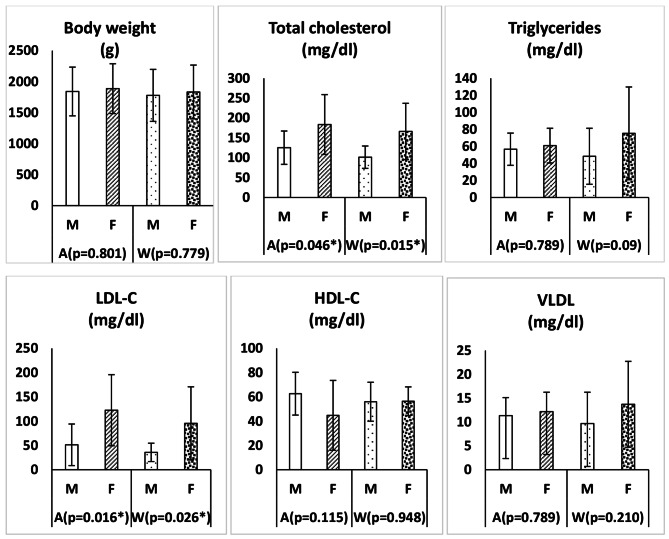
Changes in body weight and serum lipid profile (mean ± SD) in clinically healthy adult Zarudny’s spur-thighed tortoises depending on the gender and season M: male; F: female; A: autumn; W: winter; *: Significant

**Fig. 2:**
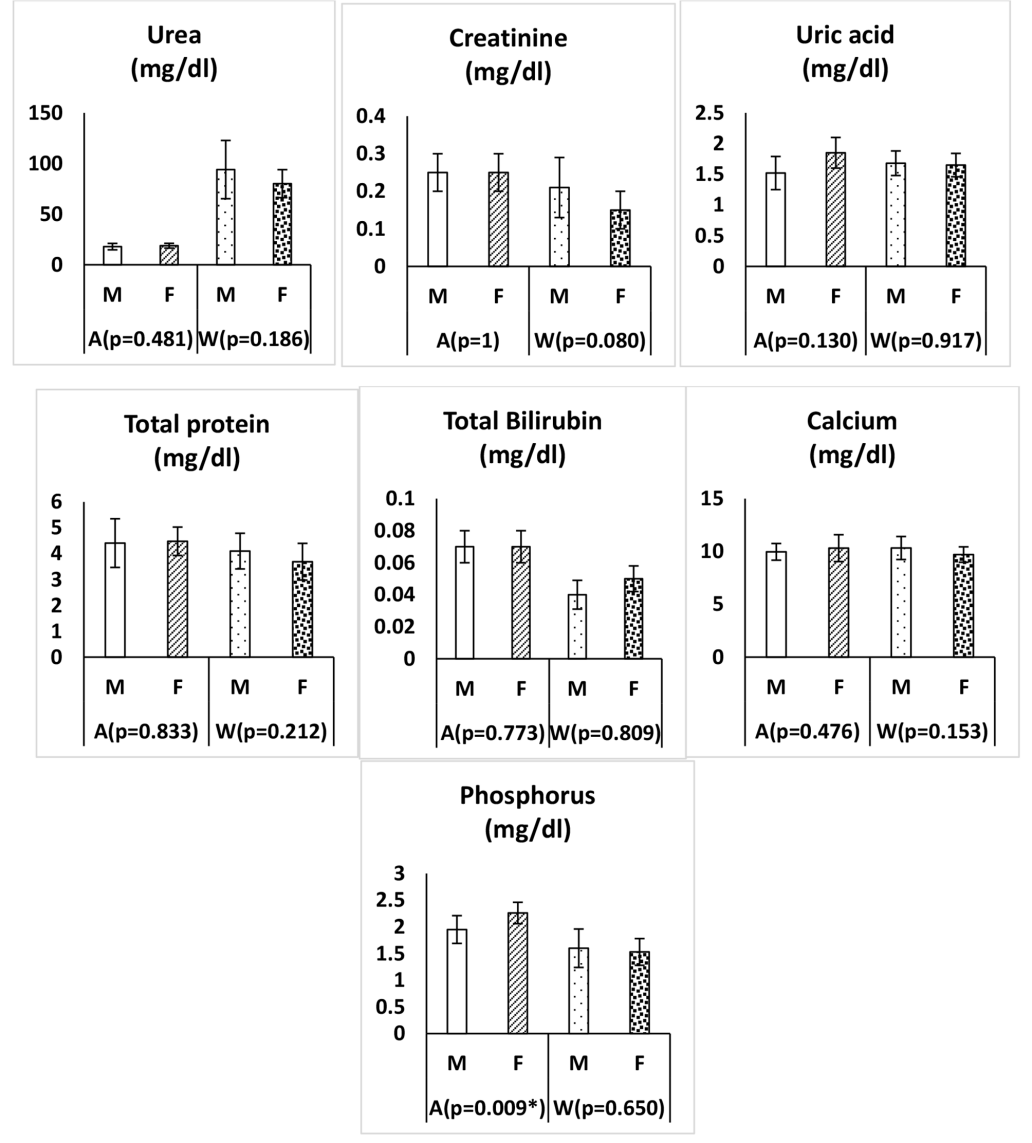
Changes of some serum biochemical parameters (mean ± SD) in clinically healthy adult Zarudny’s spur-thighed tortoises depending on the gender and season M: male; F: female; A: autumn; W: winter; *: Significant

**Fig. 3:**
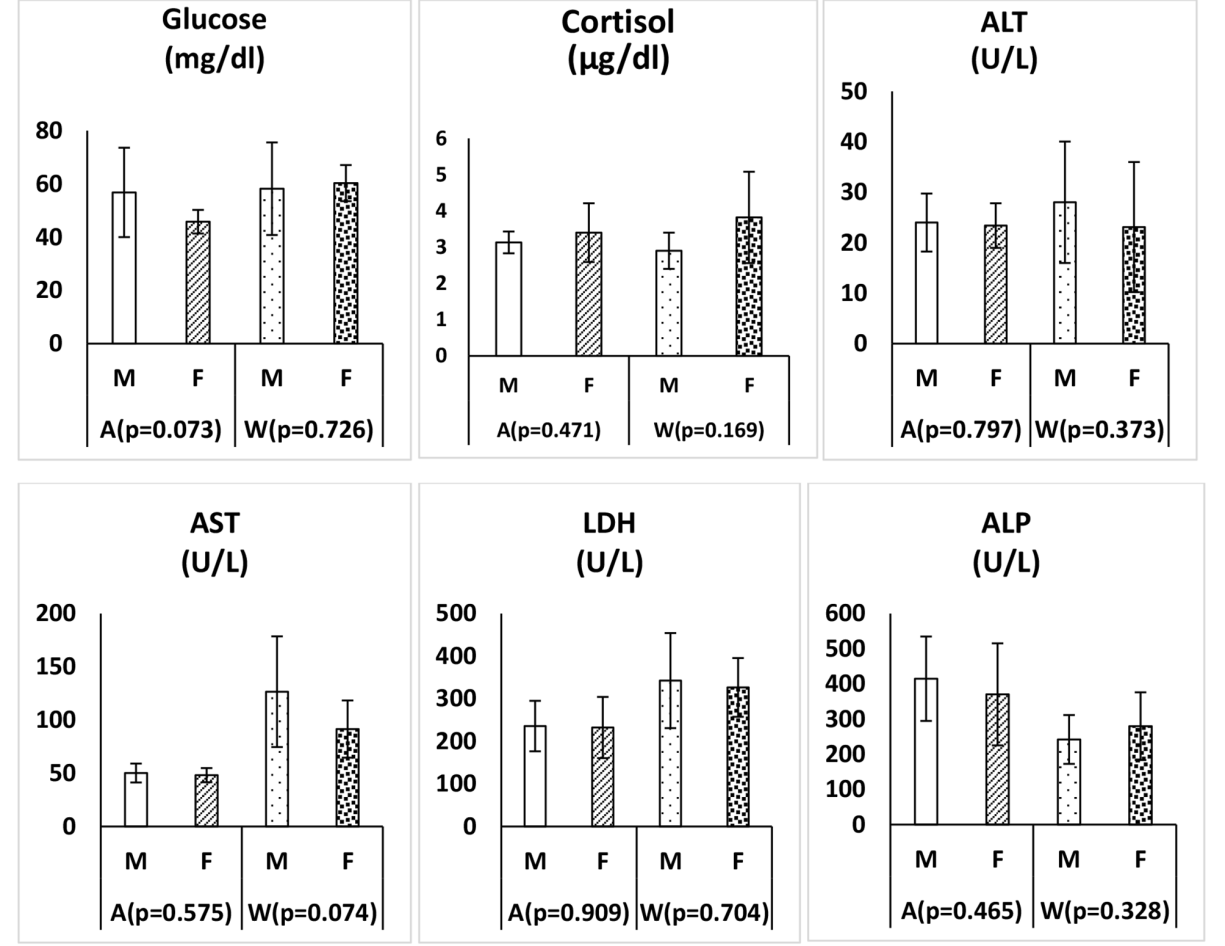
Changes in serum glucose, cortisol and enzyme activities (mean ± SD) in clinically healthy adult Zarudny’s spur-thighed tortoises depending on the gender and season M: male; F: female; A: autumn; W: winter

The changes of body weight and serum lipid profile in clinically healthy adult Zarudny’s spur-thighed tortoises depending on the gender and season are shown in Fig. 1. Figure 2 shows the changes of some serum biochemical parameters in clinically healthy adult Zarudny’s spur-thighed tortoises depending on the gender and season. In addition, the changes in serum glucose, cortisol and enzyme activities in clinically healthy adult tortoises depending on the gender and season are shown in Fig. 3. Body weight was higher in females than males in both seasons, but this difference was not significant (*P* > 0.05). The weight loss of tortoises during hibernation was 3.90 ± 0.59% (range 0.0to 10.10%). However, there was no significant difference in the weight loss of males and females (*P* = 0.971).

Cholesterol levels in females were significantly higher than males in autumn (*p* = 0.046) and winter (*p* = 0.015). Also, females had higher LDL-C levels in autumn (*p* = 0.016) and winter (*p* = 0.026). Although the mean concentration of triglyceride and VLDL in female tortoises was higher than that of males in both seasons, no significant difference was observed.

The concentration of inorganic phosphorous in females was significantly higher than that of males only before hibernation (autumn) (*p* = 0.009) and in winter its concentration did not differ significantly by gender. There was no significant difference in calcium concentration according to season and gender. However, female tortoises had lower calcium concentrations during hibernation. There was also no significant difference in concentration of other biochemical parameters between sexes in autumn and winter.

## Discussion

The spur-thighed tortoises are endangered and vulnerable [5]. Environmental policies should be adopted to prevent their extinction. Serum biochemical evaluations are one of the less invasive methods that can be easily implemented and can probably help in environmental policies to monitor the health of these tortoises and distinguish pathological from physiological processes [8].

Our finding showed that the mean weight loss of tortoises during hibernation was 3.90 ± 0.59%. Therefore, it seems that the tortoises in our study were in good condition before hibernation. A similar result was reported in a study on *Testudo graeca* during hibernation [16], while another study showed that more weight loss (mean decrease of 20%) occurred during hibernation in *Testudo hermanni hermanni* [17]. However, severe weight loss can indicate that the tortoises were in poor condition before hibernation [16]. Severe weight loss can also indicate problems in the state of health, such as liver or kidney diseases, or errors in hibernation, such as incorrect temperatures and humidity. In our study, although body weight was not significantly different by gender, it was higher in females than males in both seasons. However, the females are usually heavier than males of the same age, which may be related to physiological activities and reproduction [18].

The plasma lipoproteins of turtles are very similar to humans in both protein and lipid composition [19], and probably have similar functions [20]. They are categorized by their density characteristics including VLDL, LDL-C and HDL-C [15]. The main function of the HDL-C fraction is the transport of cholesterol, while the transport of triglycerides is mainly carried out by the LDL-C and VLDL fractions. In the present study, the reduction of cholesterol and LDL-C concentrations in winter was not significant compared to autumn. However, non-avian reptiles can drastically reduce their metabolism during prolonged starvation [19], and some reptiles do not show seasonal changes in fat stores [21].

However, high concentrations of cholesterol, LDL-C and phosphorus in female tortoises in autumn were consistent with vitellogenesis and egg production. The concentration of plasma lipids increased in late summer and early autumn along with follicular growth and vitellogenesis [22]. Although, Lance et al. [23] described that in female desert tortoises, seasonal changes in lipid concentrations appear to be driven by changes in plasma estradiol, the physiological connection between lipid (cholesterol and triglycerides) and estradiol in egg-laying species (especially during hibernation), is not as well understood as mammals.

Although, in our study, the serum triglyceride levels of some female tortoises were higher than those of males in autumn and winter, no significant changes were observed in mean triglycerides values of females in two seasons. However, Christopher et al. stated that in desert tortoises, in addition to cholesterol, triglycerides were significantly higher in females than in males [12], which is not consistent with our results.

Asadi et al. described that the Central Asian tortoise (Agrionemys horsfieldii) had the highest level of cholesterol in the LDL-C in prehibernation [24]. In our study, the amounts of cholesterol and LDL-C in the females are higher than the males in autumn and winter. The increase of cholesterol levels in females in autumn and winter was 0.51 and 0.6 times that of males, respectively. Also, serum LDL-C concentrations in females increased by 2.4 and 3 times compared to males in autumn and winter, respectively. Therefore, it seems that LDL-C fraction is the major carrier of cholesterol in female spur-thighed tortoises.

In our study, during hibernation, adult tortoises had lower serum total protein, inorganic phosphorus, creatinine, and total bilirubin, consistent with decreased nutrient intake. In addition, a remarkable increase in serum urea was found in tortoises. One of the important sources of energy during hibernation is endogenous protein degradation, which is reflected by the increased blood urea levels [16], and decreased total protein at late hibernation [12]. However, protein catabolism may not have an apparent effect on body weight [12]. In our study, serum urea levels were high (approximately 4.7-fold) in all tortoises at emergence from hibernation. These changes were similar to those previously described for desert tortoises [12]. Christopher et al. reported that male desert tortoises tended to have slightly higher urea concentrations than females [12], which is consistent with our results.

Uric acid is the primary catabolic end product of nitrogen in terrestrial reptiles. Increased plasma uric acid is not specific or sensitive for renal disease in terrestrial reptiles [13]. During hibernation, tortoises store uric acid and urea (nitrogenous wastes) in their bladders, and increase the osmolality of urine [25]. Urea concentration gradually increases in urine during hibernation. Because urea can passively diffuse across the bladder wall, an increase in urinary urea ultimately leads to an increase in blood urea [26]. High urea levels are also considered a mechanism by which plasma osmolarity is raised to reduce water loss from the body or to decrease the freezing point temperature [8]. Previous studies have shown that plasma UA concentrations, were significantly higher after hibernation in Hermann’s tortoises [8, 27, 28] and in desert tortoises [12]. In our study, although, the highest measured uric acid concentration was 3.1 mg/dl during hibernation, the increase of uric acid was not observed in all tortoises. Therefore, unlike urea, the mean serum uric acid concentration did not increase significantly during hibernation compared to autumn, which can indicate that the osmotic function of urea is higher than that of uric acid. However, females had an increase in serum uric acid levels in the autumn, which was described by Leineweber et al. [11] in Hermann’s tortoises. The reason for higher uric acid in the serum of females compared to males is not known and needs further research [27].

In Hermann’s tortoises, Scope et al. [8] stated that there was no significant difference in calcium concentration before and after hibernation in males and females. Although, our results showed that the changes of calcium concentration in both seasons and sexes were not significant, the mean calcium concentration in females was lower than males during hibernation, which is consistent with the observation of Christopher et al. [12] in desert tortoises. However, they reported that calcium levels were significantly seasonal only in females. Since some of the calcium is bound to protein, protein reduction may have contributed to calcium reduction [12].

The blood glucose level of normal reptiles varies with nutritional status, environmental conditions, and species [13]. It has previously been described that glucose increased significantly at the time of arousal from hibernation [12, 16]. It seems that gluconeogenesis and glycogenolysis may play a role in the emergence of tortoises from hibernation [16]. In our study, the difference in mean glucose in winter compared to autumn was not significant because the increase in glucose values in winter did not occur in all tortoises and not all of them emerged from hibernation at the same time.

Cortisol plays an important role in the stress response in human and domestic animals and cortisol also increases blood sugar by releasing stored glucose [15]. However, how turtles respond to stress is still unknown. In our research, there was no significant difference in serum cortisol concentration between seasons. Similar to glucose, increased cortisol concentrations did not occur in all tortoises during hibernation, which could be due to individual characteristics [15].

Plasma AST, LDH, ALT and ALP activities are not considered to be organ specific and have a wide tissue distribution in reptiles [13], and the tissue origin of the mentioned enzymes in adult spur-thighed (*Testudo graeca*) tortoises remains unknown [15]. In our research, although no significant changes were observed in serum ALT activity values according to season and gender, serum ALP activity was significantly lower in winter than in autumn which is consistent with other studies on desert tortoises [12, 28]. However, the changes of ALP in males and females were not significant. Regarding AST and LDH activities, the present study showed an increase in AST and LDH in winter compared to autumn. Although, decreased metabolic activity during hibernation was demonstrated by low plasma enzyme activity in desert tortoises [12], in a study in Hermann’s turtles, it was stated that the increased activities of AST and LDH in different seasons are probably caused by physiological changes in the liver rather than cellular damage [8]. Although, the tortoises in the present study were in good condition, the fact that the increase in serum AST and LDH at or just prior to emergence from hibernation was due to metabolic changes or liver damage/muscle degeneration requires more research.

The limitation of our study was the evaluation of the serum biochemical parameters in Zarudny’s spur-thighed tortoises in two seasons. However, more studies are needed to evaluate blood biochemical values in other seasons.

## Conclusions

The results of this study demonstrate hibernation effects on some important blood biochemical parameters compared to the autumn in the adult Zarudny’s spur-thighed tortoises. Therefore, seasonal and sex-specific reference intervals should be used for biochemical parameters in this valuable species. The present study is the first study that monitors serum biochemical parameters of adult Zarudny’s spur-thighed tortoises (*Testudo graeca zarudnyi*) according to season (autumn and winter) and gender.

## Data Availability

The data used and/or analyzed during the current study are available from the corresponding author on reasonable request.

## Abbreviations

IUCN: International Union for Conservation of Nature
LDL-C: Low-density lipoprotein cholesterol
HDL-C: High-density lipoprotein cholesterol
VLDL: Very low-density lipoprotein
ALT: Alanine aminotransferase
AST: Aspartate aminotransferase
ALP: Alkaline phosphatase
LDH: Lactate dehydrogenase
SE: Standard error
